# Optical coherence tomography (OCT) and OCT angiography: Technological development and applications in brain science

**DOI:** 10.7150/thno.97192

**Published:** 2025-01-01

**Authors:** Luyao Yang, Pengyu Chen, Xiaofei Wen, Qingliang Zhao

**Affiliations:** School of Pen-Tung Sah Institute of Micro-Nano Science and Technology, State Key Laboratory of Vaccines for Infectious Diseases, Xiang An Biomedicine Laboratory, Center for Molecular Imaging and Translational Medicine, Department of Vascular & Tumor Interventional Radiology, The First Affiliated Hospital of Xiamen University, School of Medicine, School of Public Health, Xiamen University, Xiamen 361102, China.

**Keywords:** optical coherence tomography, brain cancer, ischemic stroke, traumatic brain injury

## Abstract

Brain diseases are a leading cause of disability and death worldwide. Early detection can lead to earlier intervention and better outcomes for patients. In recent years, optical coherence tomography (OCT) and OCT angiography (OCTA) imaging have been widely used in stroke, traumatic brain injury (TBI), and brain cancer due to their advantages of *in vivo*, unlabeled, and high-resolution 3D microvessel imaging at the capillary resolution level. This review summarizes recent advances and challenges in living brain imaging using OCT/OCTA, including technique modality, types of diseases, and theoretical approach. Although there may still be many limitations, with the development of lasers and the advances in artificial intelligence are expected to enable accurate detection of deep cerebral hemodynamics and guide intraoperative tumor resection *in vivo* in the future.

## 1. Introduction

Brain disorders are diseases that affect the functioning of the human brain and neural system, including stroke, traumatic brain injury and cancer. These diseases have a significant negative impact on the physical and mental health of individuals and cause a substantial socio-economic burden on society.

Various factors can cause or worsen brain symptoms. Due to the aging of population and changing lifestyles, the incidence of the brain disorders is increasing globally. According to the World Health Organization, the stroke is responsible for approximately 52 million deaths annually, making it one of the leading causes of human death and disability. In addition, modern lifestyle and environmental factors are also linked to an increased risk of brain diseases. For instance, brain disorders are associated with chronic sleep deprivation, poor dietary habits, high-pressure jobs, stress, smoking, and alcohol abuse. The incidence of brain disorders continues to increase globally due to these multiple causes.

Currently, computed tomography angiography (CTA) 1-3, magnetic resonance angiography (MRA) 4-6, digital subtraction angiography (DSA), and ultrasound imaging (US) 7-9 are primary modalities for monitoring and investigating brain diseases. However, these imaging techniques are associated with inherent limitations and shortcomings. CTA delivers high-quality three-dimensional images to detect stenoses, tumors, and other cerebral artery and vein lesions. Nevertheless, CTA is associated with potential risks such as radiation exposure and contrast allergies. DSA employs contrast agents and X-rays to delineate blood vessel structure and hemodynamics, precisely visualizing vascular abnormalities. However, DSA poses significant risks of contrast agent dosage and radiation exposure. MRA uses magnetic and pulsed magnetic fields to generate images without contrast and radiation, thus enhancing safety. However, MRA is less proficient than CTA in showing small vessels and details. The US is a non-invasive imaging modality that employs sound waves to visualize blood vessels with high resolution. However, it is limited in its ability to visualize the skull and deep anatomical structures.

In addition, many optical techniques are also widely used in medical imaging. For example, photoacoustic imaging utilizes the interaction of optics and acoustics 10. By irradiating a sample with a laser light pulse, the sample absorbs the light energy and produces transient thermal expansion, which in turn causes the emission and detection of acoustic waves, thus enabling imaging of tissue structure and function. It enables deep penetration of biological tissues, but at the same time sacrifices a certain imaging resolution. Multiphoton imaging utilizes the non-linear optical process of multi-photon excitation to achieve high-resolution imaging of samples 11. However, it is more demanding on the laser, and fluorescence may have certain side effects. Speckle imaging is used to image a sample by observing a scattering pattern due to the coherence of light, and can be used to measure the deformation or motion of a sample 12. However, the resolution of Speckle imaging is relatively low and motion artefacts are more obvious. Some other imaging techniques, such as light sheet microscopy and expansion microscopy, require rigorous sample preparation process 13, 14.

**Figure 1:**
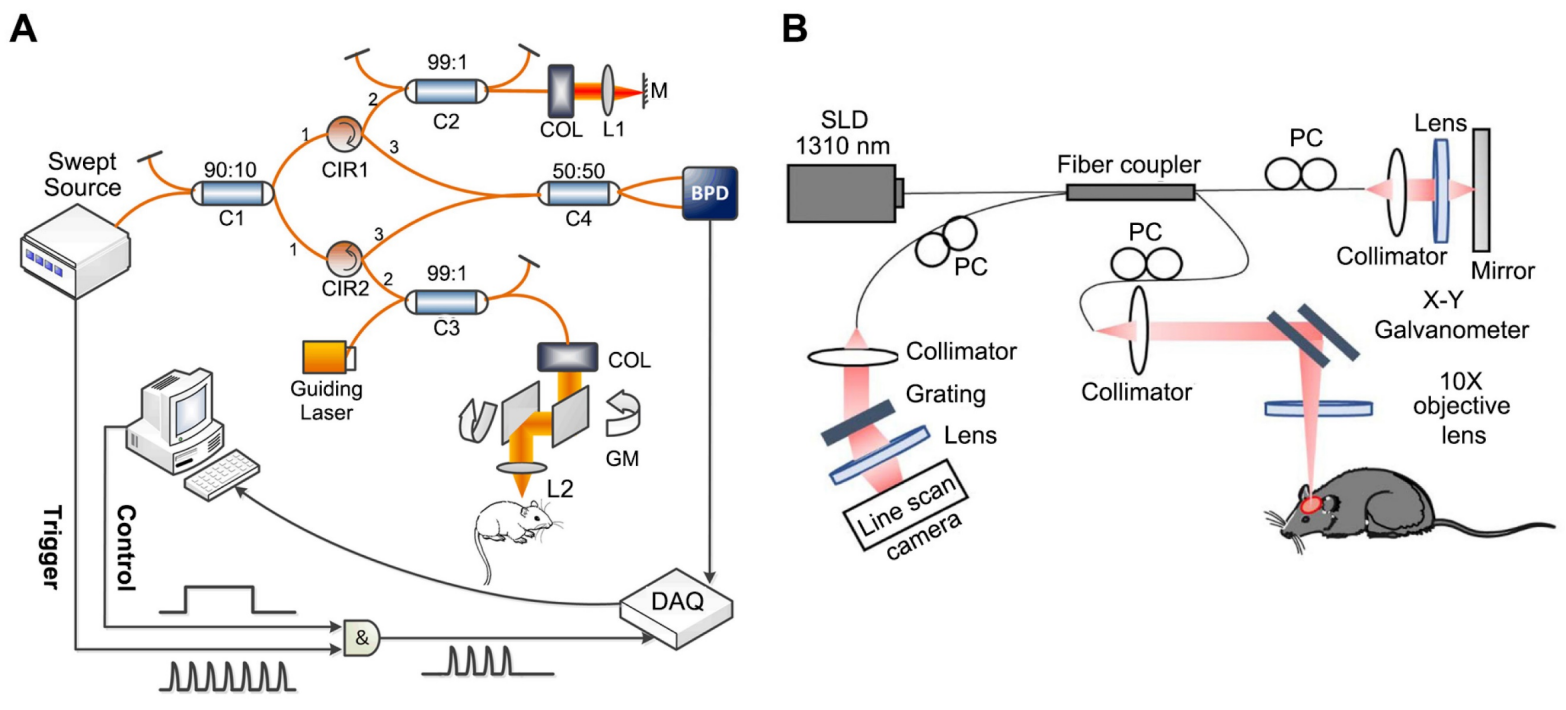
**(A)** The implementation of an SS-OCT system. Reproduced with permission from 17, copyright 2019, SPIE. **(B)** The implementation of an SD-OCT system. Reproduced with permission from 18, copyright 2021, Elsevier.

In recent years, optical coherence tomography (OCT) has demonstrated advantages in various research areas, especially examining eye diseases. OCT based on the principle of low coherence interference and generates cross-sectional (2D) or three-dimensional (3D) images by measuring the magnitude and time delay of the backscattered light from the sample, which is classified into time-domain OCT (TD-OCT) and frequency-domain OCT (FD-OCT) according to the imaging principle. As early OCT systems, TD-OCT comprise an interferometer featuring a light source with low coherence and broad bandwidth 15. For FD-OCT, its light source and detector type are divided into spectral-domain OCT (SD-OCT) and swept-source OCT (SS-OCT). In SD-OCT, a broad-bandwidth light source and a spectrometer are utilized for signal detection, whereas SS-OCT obtains depth-resolved tissue information by sweeping a range of optical frequencies, with spectral interferograms typically detected by a photodiode detector 16. Figure 1 shows the setup of the SS-OCT system 17 and SD-OCT 18.

Unlike Magnetic Resonance Imaging (MRI) or computed tomography (CT), OCT does not require injections or expose patients to ionizing radiation, making it a safer imaging choice, particularly for individuals sensitive to contrast agents or with specific health conditions. In addition, OCT has a higher resolution than other imaging modalities and allows for real-time, non-invasive imaging. Thus, OCT presents a novel approach for imaging brain diseases as a high-resolution, non-invasive diagnostic tool 19, 20. The high resolution of OCT enables the acquisition of detailed data on diverse brain structures.

Optical coherence tomography angiography (OCTA), an advanced OCT variant, has seen rapid advancement in recent years. OCTA differentiates between moving particles and static tissue by analyzing variations in OCT signals from the same location at different times, enabling the visualization of microvascular networks in biological tissues without requiring dye injections. Technological advancements are poised to enhance OCT-based blood flow imaging technologies, yielding faster and higher quality images 21,22. These developments enable researchers and medical professionals to conduct detailed brain examinations crucial for early disease detection.

**Figure 2:**
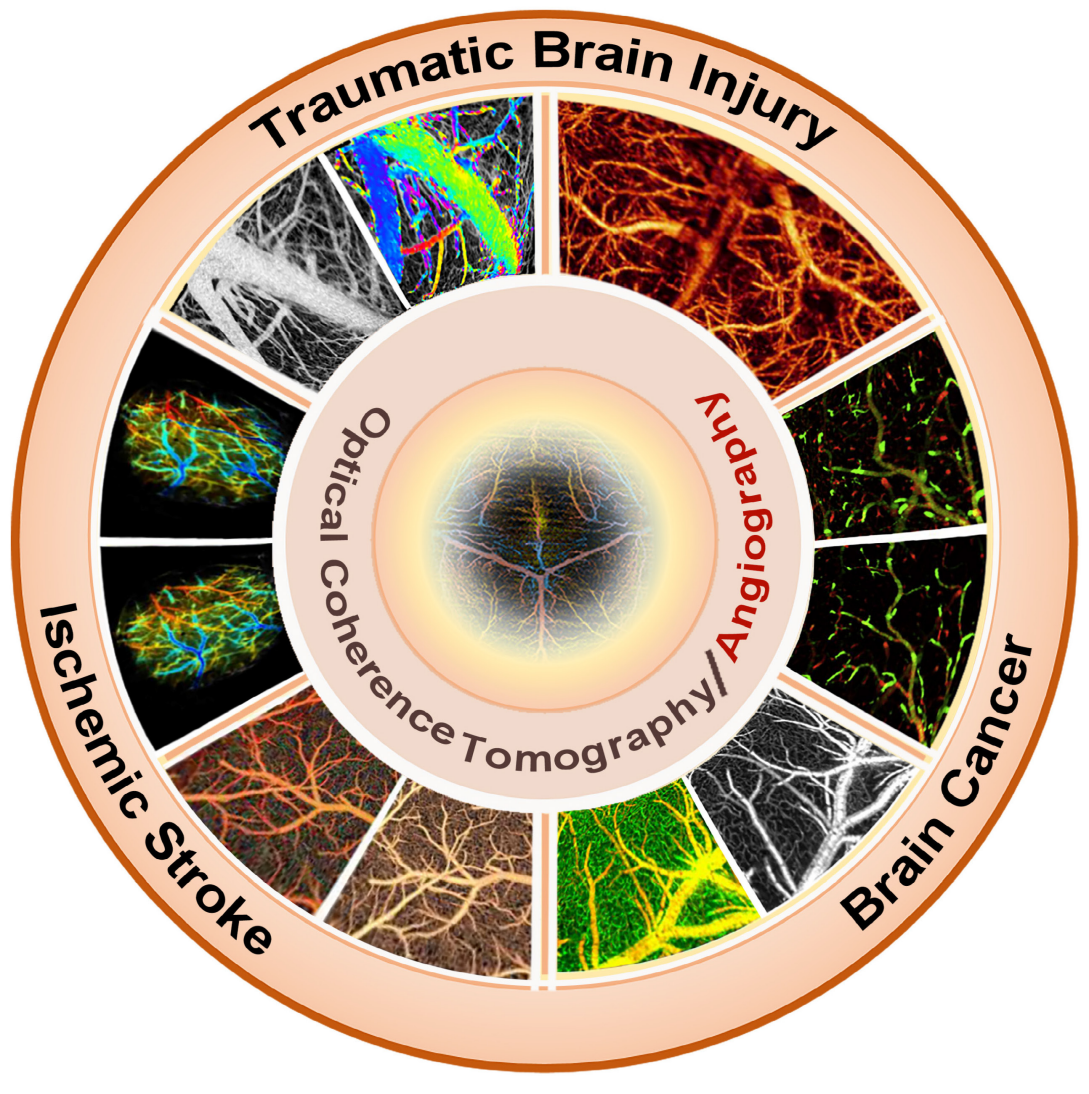
OCT cerebral vascular imaging and its application. Reproduced with permission from 18, copyright 2021, Elsevier. Reproduced with permission from 22, copyright 2016, Elsevier. Reproduced with permission from 30, copyright 2021, SPIE. Reproduced with permission from 33, copyright 2016 Elsevier. Reproduced with permission from 78, copyright 2013 PLoS One. Reproduced with permission from 94, copyright 2009 SPIE.

OCT imaging is increasingly applied to various brain disease models, including stroke, brain injury, brain cancer, and others (Table 1). Studies indicated that OCT can identify structural and vascular changes in the brain at an early stage of symptom onset (**Figure 2**). Timely detection through OCT could facilitate prompt intervention and enhance patient treatment outcomes. Moreover, the high-resolution images generated by OCT may serve as a valuable resource for guiding surgical interventions, such as the excision of brain tumors. Furthermore, OCT holds promise in monitoring the efficacy of treatments for neurological disorders. This review aims to consolidate current research advancements utilizing OCT in analyzing the brain cortex and various brain pathologies, highlighting OCT's robust diagnostic potential for research in brain diseases alongside its clinical implications and prospects for the future.

## Applications of OCT Imaging in the Brain

The brain is the most important part of the vertebrate central nervous system and encompasses a wide area including the cerebral blood vessels and brain parenchyma. It is responsible for influencing and regulating other tissues and organs of the body to maintain life functions. The large number of organized cerebral blood vessels ensures sufficient blood flow to supply the brain with oxygen and nutrients 23. By regulating the blood flow of each vessel, the blood perfusion of the brain is guaranteed, the brain can have a certain self-regulation ability to the disease. In addition, the nerve cells and glial cells in the brain parenchyma ensure the transmission and processing of information in the brain, regulating vital activity, mental activity, and sensorimotor activity 24. When abnormalities occur in the blood vessels and tissues of the brain, it can lead to a range of brain disorders, including stroke and brain cancer 25.

Numerous researchers in neuroscience have been exploring OCT imaging systems that utilize angiography and Doppler effects to examine cerebrovascular and cerebral hemodynamics. Maheswari *et al.* initially illustrated the successful application of OCT techniques in studying brain function 26. However, the current optical imaging methods face significant limitations in generating cerebrovascular imaging and cerebral hemodynamics in tissue beds due to the tissue's high scattering and absorption of light.

**Figure 3:**
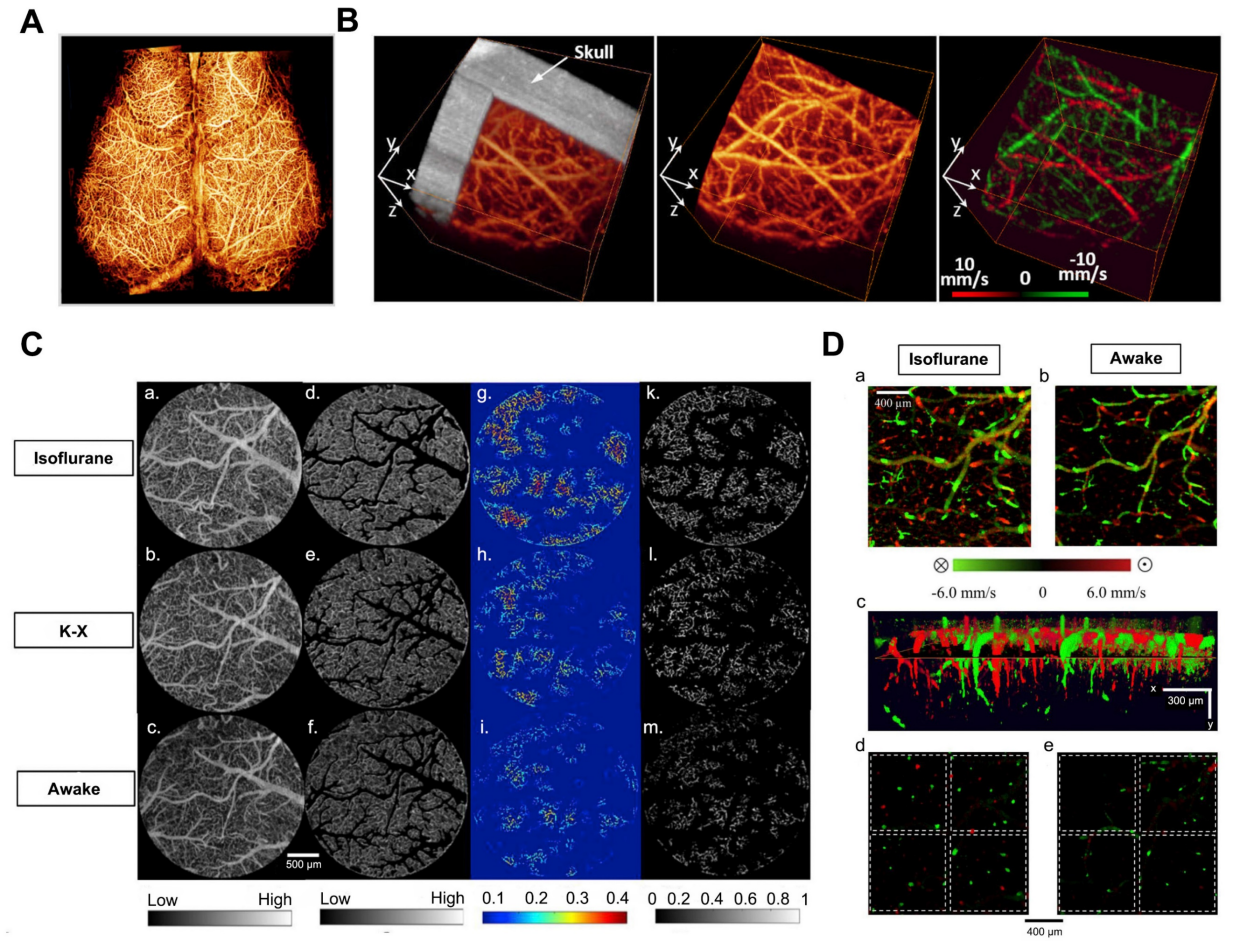
** (A)** UHS-OMAG imaged functional blood flow networks throughout the cerebral cortex of mice *in vivo*. Reproduced with permission from 28, copyright 2009, Elsevier. **(B)** *In vivo* 3D OMAG imaging of the cortical brain of a mouse with the skull left intact. Reproduced with permission from 29, copyright 2010, Optica Publishing Group. **(C)** Comparison of VAD and flux in capillaries: (a-c) OMAG angiograms corresponding to isoflurane, ketamine-xylazine, and awake states. (d-f) OMAG angiograms with arteries and veins excluded. (g-i) Capillary VAD maps at three states. (k-m) CFI maps at three states.** (D)** Comparison of CBF parameters in one animal. (a-b) Bidirectional axial CBF velocity maps of mouse cortex at isoflurane and awake regime. (c) 3D visualization with descending and ascending vessels. (d-e) Orthogonal slices below the cortical surface at isoflurane and awake regime. Reproduced with permission from 18, copyright 2021, Elsevier.

To overcome these limitations, Wang *et al.* developed an optical angiography (OAG) method for high-resolution imaging of the cerebral cortex in minutes without the need for dye injection, contrast agents, or craniotomy 27. Subsequently, they introduced the optical microvascular angiography (OMAG) technique and the Doppler optical microvascular angiography (DOMAG) method, which demonstrated the capability to attain capillary-level resolution in imaging the meninges and cortical vasculature (Figure 3A) 28, as well as to visualize blood flow velocities in functional vessels within microcirculatory tissue beds in real-time (Figure 3B) 29. A recent study integrated the OMAG and DOMAG techniques to compare vascular flow parameters in the mouse cortex between states of anesthesia and wakefulness 18. The evaluation of cerebral blood flow (CBF) involved systematic measurements of changes in axial flow velocity and total blood flow (Figure 3C), vessel area density (VAD), capillary flux index (CFI), artery and vein diameters (Figure 3D).

**Figure 4:**
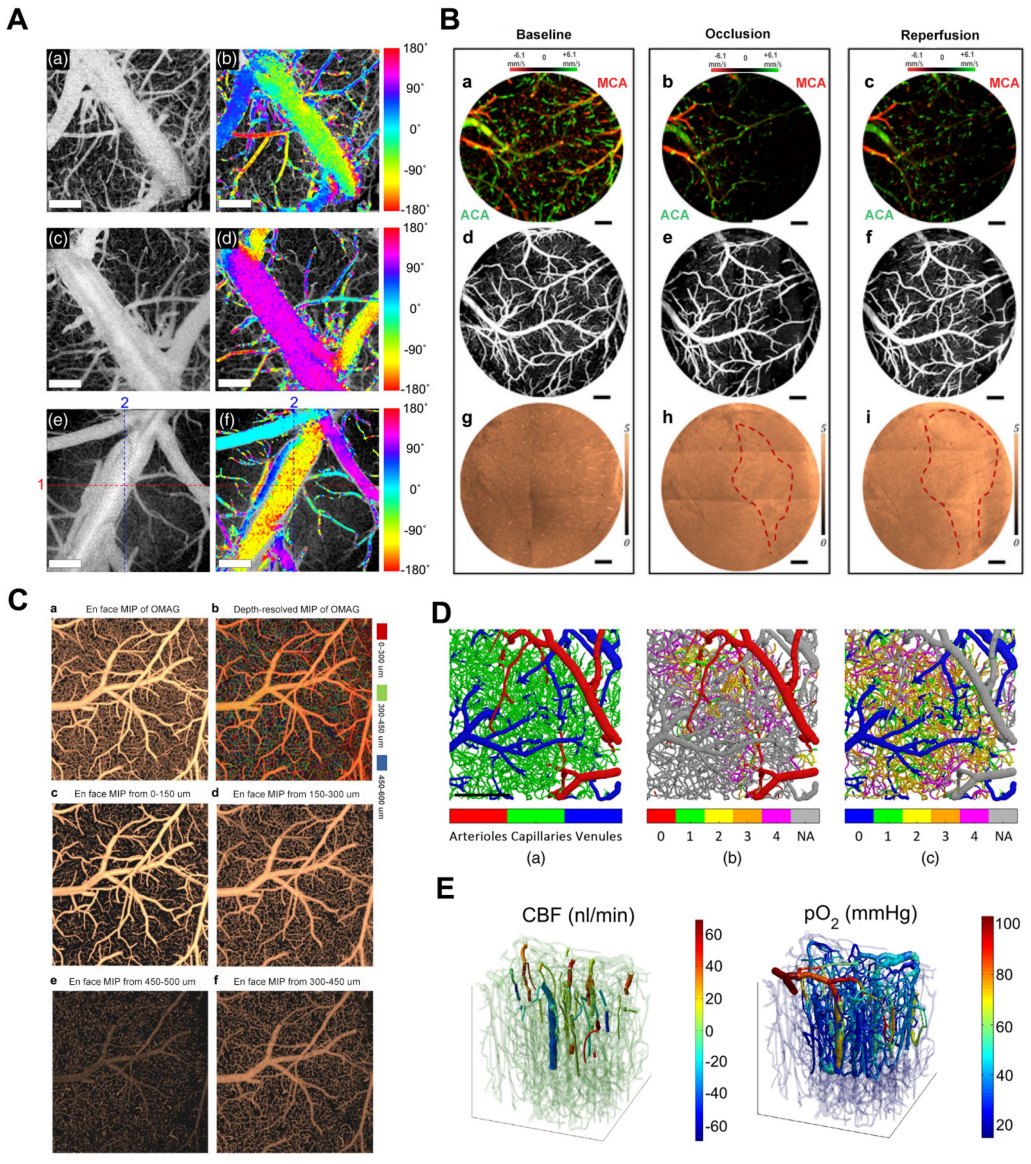
** (A)** *In vivo* 2D transverse flow direction images of mouse brains. (a), (c), and (e) En face mean projections of the inter B-scan OCTA volume images. (b), (d), and (f) Color-encoded blood flow direction information overlaid on the corresponding en-face OCTA images. Reproduced with permission from 30, copyright 2021, SPIE. **(B)** The TIM of a 60D dataset on mouse cerebral cortex under baseline, 3-min MCA occlusion, and reperfusion conditions through a cranial window. (a-c) 0 - 500 μm depth axial velocity distribution of the face MIP. (d-f) 0 - 100 μm depth of the microcirculation network of the face MIP. (g-i) En face sAIP of OAC image. Red dashed line points out the region of variation in tissue scattering properties. Reproduced with permission from 32, copyright 2015 Optica Publishing Group. **(C)** The frontal classified maximum intensity projection (sMIP) images of the segmented OMAG data. (a) Frontal sMIP images of OMAG at 0-600 μm depth. (b) Frontal depth-resolved (color-coded) sMIP images of OMAG at 0-600 μm depth. (c-f) Frontal sMIP images of OMAG at different depths. Reproduced with permission from 33, copyright 2016 Elsevier. **(D)** Angiographic segmentation and superposition of vessel types. Reproduced with permission from 51, copyright 2023, SPIE. **(E)** Co-registration of the Two-Photon Microscopy and OCT data from the upper 650 um in a mouse somatosensory cortex. Reproduced with permission from 53, copyright 2015, Biomed Opt Express.

Shin *et al.* presented an OCTA imaging technique to visualize two-dimensional (2D) lateral blood flow direction, enabling the observation of blood flow direction within the cerebral vascular network of live mice (Figure 4A) 30. Baran *et al.* combined the OAC reconstruction method developed by Vermeer *et al.* recently with OMAG to achieve more precise tissue injury mapping (TIM) 31. TIM utilizes a non-invasive *in vivo* optical coherence tomography method to generate light attenuation coefficients and microvascular maps of damaged tissue (Figure 4B) 32. TIM visualized the development of infarcted regions in the mouse cerebral cortex during stroke. Baran further developed image processing methods to accurately distinguish the window and skull from the cortex and precisely segment the distinct layers of capillary beds (Figure 4C) 33. Merkle *et al.* introduced dynamic contrast optical coherence tomography (DyC-OCT) 22, a novel technique that employs cross-sectional imaging of intravascular tracer kinetics to measure the distribution of capillary transit times at a microscopic level, providing a new perspective of cortical microvascular networks. Subsequently, they quantified microvascular CBF and CBV within specific layers across the depth of the mouse neocortex, establishing a link between cortical vascular structure and *in vivo* brain vascular physiology 34.

Several studies have been devoted to specific methods and algorithms that enable OCT to provide quantitative and qualitative insights into the function and structure of the cerebral vasculature. Shin *et al.* introduced an OCT imaging technique to assess vasodilation propagation in response to functional congestion in conscious mice, demonstrating the potential of OCTA in measuring stimulus-induced retrograde vasodilation in awake mouse brains 35. Choi *et al.* proposed a computational method that leverages the reduced mean OCT projection intensity of penetrating vessels and the increased variance of Doppler frequency to accurately identify and grade cortical penetrating vessels in perfusion. They noted a substantial decrease in the density of penetrating vessels in a murine model of focal ischemic stroke 36. OCT has been employed in diverse brain disease models to elucidate disease pathophysiology and improve prognostic strategies 37-50.

In addition, OCT-based multimodal systems have been shown to provide more comprehensive information for cerebrovascular imaging in recent years. Optical coherence tomography and two-photon microscopy (2PM) offer distinct advantages and can image cerebral blood vessels. Pian *et al.* introduced a novel data and processing framework to synchronize various microvascular blood flow velocity measurements from dynamic light scattering optical coherence tomography (DLS-OCT) with corresponding microvascular angiography data acquired through two-photon microscopy. This framework facilitates the simulation of CBF and oxygen transport in microvascular networks within the brain, improving the understanding of microvascular blood flow regulation in the normal brain and various brain conditions (Figure 4D) 51. These findings demonstrate that two-photon and OCT imaging can complement each other in brain imaging, significantly enriching our insights into cerebral blood flow dynamics. Gagnon *et al.* combined two-photon laser scanning angiography with DOCT and applied OCT techniques to reconstruct the microvascular flow distribution in the mouse cortex 52. Yaseen *et al.* also developed a multimodal imaging system for studying multiple aspects of cerebral blood flow and metabolism in small animals (Figure 4E) 53.

The Dziennis's group designed an integrated multifunctional imaging system that included simultaneous dual-wavelength laser speckle imaging (DWLS) as a guiding tool for optical microangiography (OMAG) to investigate the vascular response in male mice with acute cerebral embolism 54. Tang *et al.* developed a versatile imaging system that combines phase-sensitive optical coherence tomography (PhS-OCT) with IOSI to detect neural responses in the mouse barrel cortex during whisker stimulation in cross-sectional directions 55. IOSI is utilized to map and identify hemodynamic response areas in the activated cortex, guiding depth-resolved OCT imaging. The research group also employed OMAG and IOSI to image the activated somatosensory cortex in the mouse brain 56, comparing the temporal distribution of the two signals to analyze changes in blood flow during functional activation at different depths. In addition, Li *et al.* introduced a novel application of an imaging system that incorporates an electrically tunable lens (ETL) and a customized spectral domain OCT (SD-OCT) for multifocal plane cerebral blood flow imaging in mouse cortex, overcoming the depth of focus (DOF) limitation of conventional OCT systems and OCT angiography (OCTA) in mouse cerebral cortex 21.

**Figure 5:**
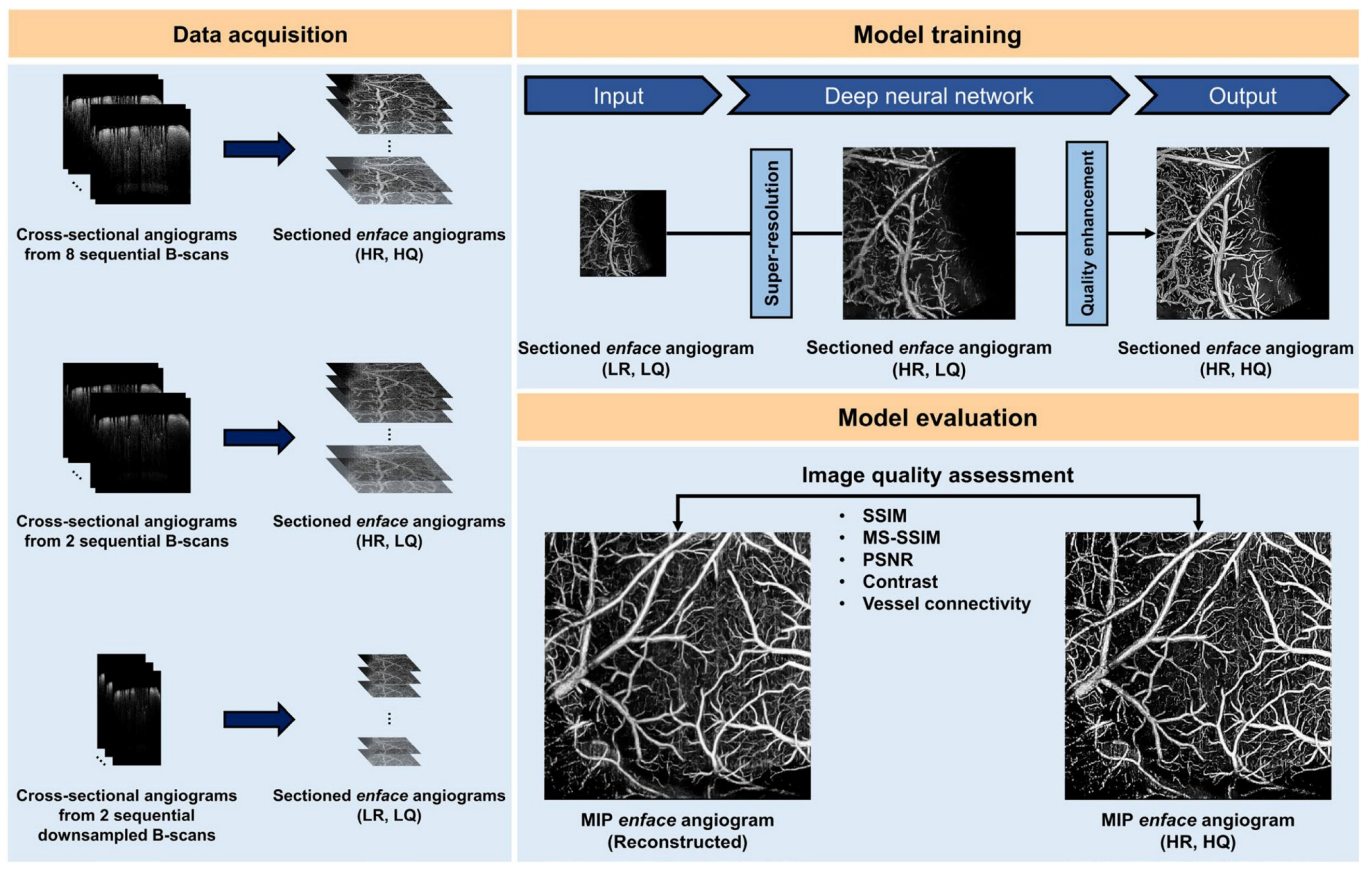
Schematic of our deep learning (DL) framework for accelerated optical coherence tomography angiography (OCTA). (LR, LQ) low-resolution and low-quality, (HR, LQ) high-resolution and low-quality, (HR, HQ) high-resolution and high quality, SSIM structural similarity index measure, MS-SSIM multiscale structural similarity index measure, PSNR peak signal-to-noise ratio. Reproduced with permission from 65, copyright 2022 nature portfolio.

Furthermore, the recent substantial progress in deep learning has prompted an increasing number of researchers to integrate OCT with deep learning 57-62. Li *et al.* introduced deep learning algorithms for segmenting and reconstructing vascular systems. The axon fiber pathways in the mouse brain were mapped using delay and optical axis direction contrast 63. Stefan *et al.* Presented an approach based on deep learning and simulations to quantify cortical capillary red blood cell (RBC) flux using optical coherence tomography (OCT) 64. Kim *et al.* developed a deep learning-based framework that enhances OCTA imaging speed without compromising image quality, offering a software-only solution that streamlines preclinical and clinical studies (Figure 5) 65. In 2023, the Pan's research group utilized ultra-high resolution optical coherence Doppler tomography (mu ODT) to 3D image CBF velocity (CBFv) dynamics on awake mice. They accomplished this by implementing self-supervised deep learning for efficient image denoising and motion artifact removal, offering insights into the effects of drugs and various disease conditions such as ischemia, tumors, and other pathologies 66. Zhang *et al.* combined non-invasive OCT technology for 3D global image acquisition with deep learn-based image processing to quantify microvascular networks in 3D *in vitro* BBB models, providing a rapid and non-invasive method for observing and quantifying various 3D *in vitro* models 67.

With the development and integration of technologies, OCT is progressively emerging as the preferred modality for functional brain imaging during brain activity or disease progression.

## OCT Imaging of Ischemic Stroke

Stroke is a leading cause of mortality globally, resulting in millions of deaths annually 68. Risk factors such as hypertension, diabetes 69, high cholesterol, obesity, smoking, and heart disease significantly increase the chances of experiencing a stroke, which can result in long-term disability or even death.

As a cerebral dysfunction resulting from cerebrovascular disease, stroke typically caused by the blockade or rupture of a cerebral artery. It is classified into two main categories: ischemic stroke, resulting from blood vessel blockage, and hemorrhagic stroke, resulting from blood vessel rupture. Hemorrhagic stroke commonly occurs due to the rupture of a cerebral artery, leading to a substantial blood influx into the brain, thereby compressing and damaging the adjacent brain tissue. Conversely, ischemic stroke is primarily caused by artery blockage, often attributed to thrombosis or atherosclerosis. Prompt treatment of ischemic strokes is crucial since brain cells can perish within moments due to a lack of oxygen and nutrients. OCT technology serves as a valuable tool in analyzing ischemic stroke by detecting and evaluating cerebrovascular intima thickness, fat deposits, and plaques, enabling early diagnosis and prevention. Subsequently, we will elaborate on the advancements and challenges associated with OCT imaging in ischemic stroke.

In ischemic stroke, the progression of brain injury post-occlusion unfolds over a timeline spanning the hyperacute (minutes), acute (hours), and chronic phases (days) 70,71. Following the occlusion, there is a significant reduction in the blood volume of the brain tissue at the center of the ischemic injury, resulting in a severe hypoxic environment that causes neuronal necrosis 72. Unlike the infarct core, the penumbra is functionally inhibited yet maintains its structural and metabolic integrity. Timely reperfusion of the penumbra region is essential to prevent permanent infarct progression. Therefore, a comprehensive comprehension and analysis of ischemic stroke hemodynamics are essential for precise therapeutic interventions during the acute phase and subsequent prognostic evaluations 73-75. In recent years, OCTA has been utilized to investigate real-time vascular dynamics to enhance comprehension of the intricate response and facilitate more effective stroke recovery mechanisms.

**Figure 6:**
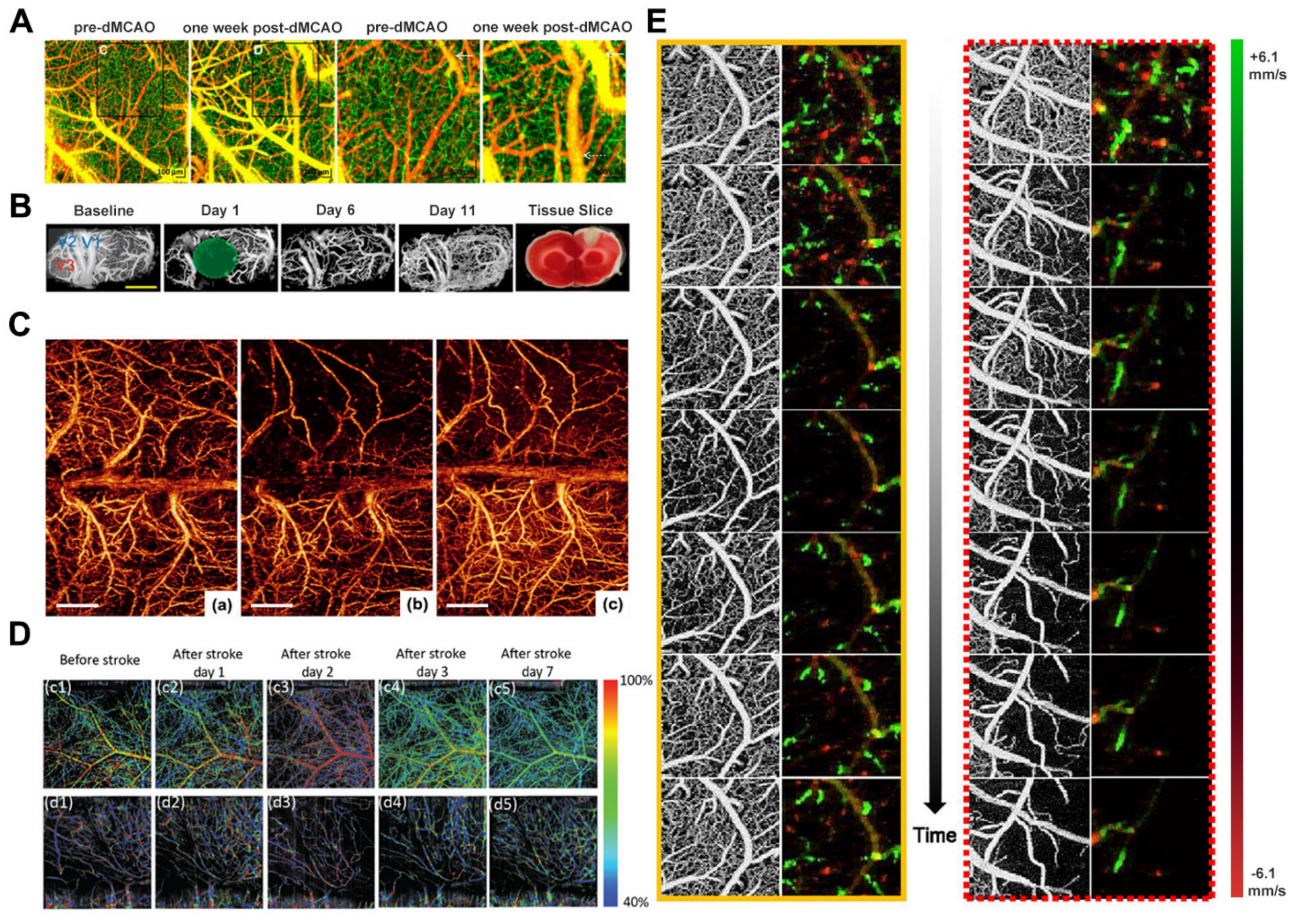
**(A)** OCT angiography shows distal boundary remodeling. Reproduced with permission from 77, copyright 2013 PLoS One. **(B)** Longitudinal monitoring of vascular response after chronic PT in male rats. Reproduced with permission from 78, copyright 2019 Sage Journals. **(C)** Comparison of 3D OMAG imaging of ischemic stroke cortex before and after MCAO and reperfusion. Reproduced with permission from 76, copyright 2011 Wiley. **(D)** Top-view and Side-view vis-OCTA en-face images pseudo-colored according to measured sO2, from immediately before stroke to 7 days after stroke. Reproduced with permission from 82, copyright 2019 Biomed Opt Express. **(E)** OMAG and DOMAG images showed dynamic changes in cerebral blood perfusion and blood flow velocity in small areas (1 mm×1 mm) proximal to ACA and MCA after dMCAO. Reproduced with permission from 81, copyright 2019 IEEE.

The predominant rodent stroke model utilized in research is middle cerebral artery occlusion (MCAO) 76. Srinivasan *et al.* designed a multiparametric OCT platform using this model for longitudinally imaging ischemic stroke in mice by surgically preparing a thin skull and enhancing cranial windows. The results demonstrated the spatio-temporal interactions between hemodynamics and cell viability (key determinants of pathogenesis) during the acute phase (**Figure 6A**) 77. Meanwhile, Yang *et al.* employed OCT to monitor dynamic changes in blood perfusion and tissue scatter in a chronic rat PT stroke model (**Figure 6B**). They use lable-free and depth-resolved OCT to reveal dynamic changes in blood perfusion and tissue scattering after ischemic stroke 78. In addition, Guo *et al.* also used OCTA to reveal the different spatiotemporal dynamics of acute, subacute, and chronic phases after ischemic stroke. They demonstrated a novel needle-shaped beam optical coherence tomography angiography (NB-OCTA) system that enables rapid imaging without scanning, which is conducive to surgical navigation and monitoring during surgery 79.

After the availability of OMAG, Jia and Wang reported the application of OMAG in a mouse ischemic stroke model (**Figure 6C**) 76. The study demonstrated the superior imaging capability of OMAG in mapping dynamic cerebral vascular perfusion with high resolution. Besides, CBF imaging eliminates the need for an "intracranial window", thereby preventing potential complications arising from factors such as brain temperature and pressure that could otherwise influence the resulting perfusion data. Kanoke *et al.* recently utilized the DOMAG technique to determine the spatiotemporal dynamics of collateral flow and downstream hemodynamics after ischemic stroke 80. The cranially intact wide-field DOMAG is well-suited for quantifying individual blood flows within 150 μm from the brain's surface in relatively large vessels, making it an excellent imaging modality to investigate dynamic changes in blood flow within leptomeningeal artery (LMA) anastomoses. This method represents a significant advancement over previous approaches for visualizing spatiotemporal blood perfusion in the post-stroke cortex and supplements other optical imaging techniques lacking flow direction data.

Choi *et al.* used a single OCT imaging platform to monitor hemodynamic and structural changes in the mouse brain during the initial three hours following stroke onset. The study employed a distal middle cerebral artery occlusion model to induce focal ischemic stroke in the mouse brain. The research gathered data on blood perfusion, velocity, flow, and light attenuation using the OCT imaging platform. These measurements offer insights into the evolution of the cortical vascular system, CBF, capillary perfusion, and tissue scatter during the acute phase of a stroke (**Figure 6E**) 81. Beckmann *et al.* introduced a vis-OCT imaging system capable of visualizing the complete cerebral cortex via a cranial window equipped with a microprism. This approach enables high-resolution, *in vivo* imaging of the mouse cortex over 60 days, offering a novel means to investigate both the superficial and deep cortical layers concurrently (**Figure 6D**) 82. Neurovascular decoupling is associated with microcirculatory dysfunction in the ischemic extra-core region after ischemic stroke. Staehr *et al.* combined OCT and laser speckle contrast imaging (LSCI) to evaluate cerebral perfusion and neurovascular coupling. Their study involved labeling lectin and platelet-derived growth factor receptor β to analyze capillaries and pericytes in perfusion-fixed tissues. The results showed that arterial occlusion resulted in pericapillary cell contraction and cessation of capillary flow within the peri-ischemic cortex. These findings suggest a potential association between capillary dysfunction and neurovascular decoupling, indicating a novel therapeutic target for further exploration 83.

## OCT Imaging of Traumatic Brain Injury

Traumatic brain injury (TBI) is an injury of the brain caused by external forces, potentially leading to the destruction or demise of brain cells, ultimately impacting brain function. This specific condition can be instigated by violence, accidents, war, sports-related incidents, and explosions 76. Global statistics estimate that around 10,000 individuals succumb to TBI annually 84. TBI stands prominently as a leading cause of mortality in both intensive care units and emergency departments. While mild cases of TBI typically do not result in enduring brain impairments, they may still influence certain cognitive and emotional capacities 85. Moderate and severe TBI can result in enduring brain damage and functional disability. In certain instances, TBI may precipitate severe complications, including coma, stroke, seizures, and hydrocephalus.

Currently, the main imaging modalities for TBI are mainly CT and MRI 84-87. However, CT scans and X-rays expose the body to radiation, limiting their frequency, whereas MRI examinations are cost-prohibitive and not conducive to routine use. In contrast, OCT offers notable advantages. Firstly, its imaging resolution is exceptionally detailed, enabling clear visualization of intricate brain tissue structures, particularly in minuscule areas of injury. Secondly, OCT is rapid, generating high-resolution images within seconds, making it invaluable for emergency diagnoses. Moreover, OCT imaging obviates the need for contrast or radioactive substances, rendering it safe for reuse without risking patient or practitioner harm. Consequently, OCT emerges as a promising tool for investigating traumatic brain injuries.

In recent years, numerous TBI models have been created to mimic the impact of injuries on neural tissue and to advance novel diagnostic and therapeutic strategies 88-91. These models encompass a range of methodologies, such as electrophysiological and imaging techniques, and are predominantly classified as focal or diffuse injuries. Research typically emphasizes focal models for accurate insight into the acute progression of tissue scarring. In contrast, diffuse injuries concentrate on long-term consequences and the subsequent injury cascade responses 92.

**Figure 7:**
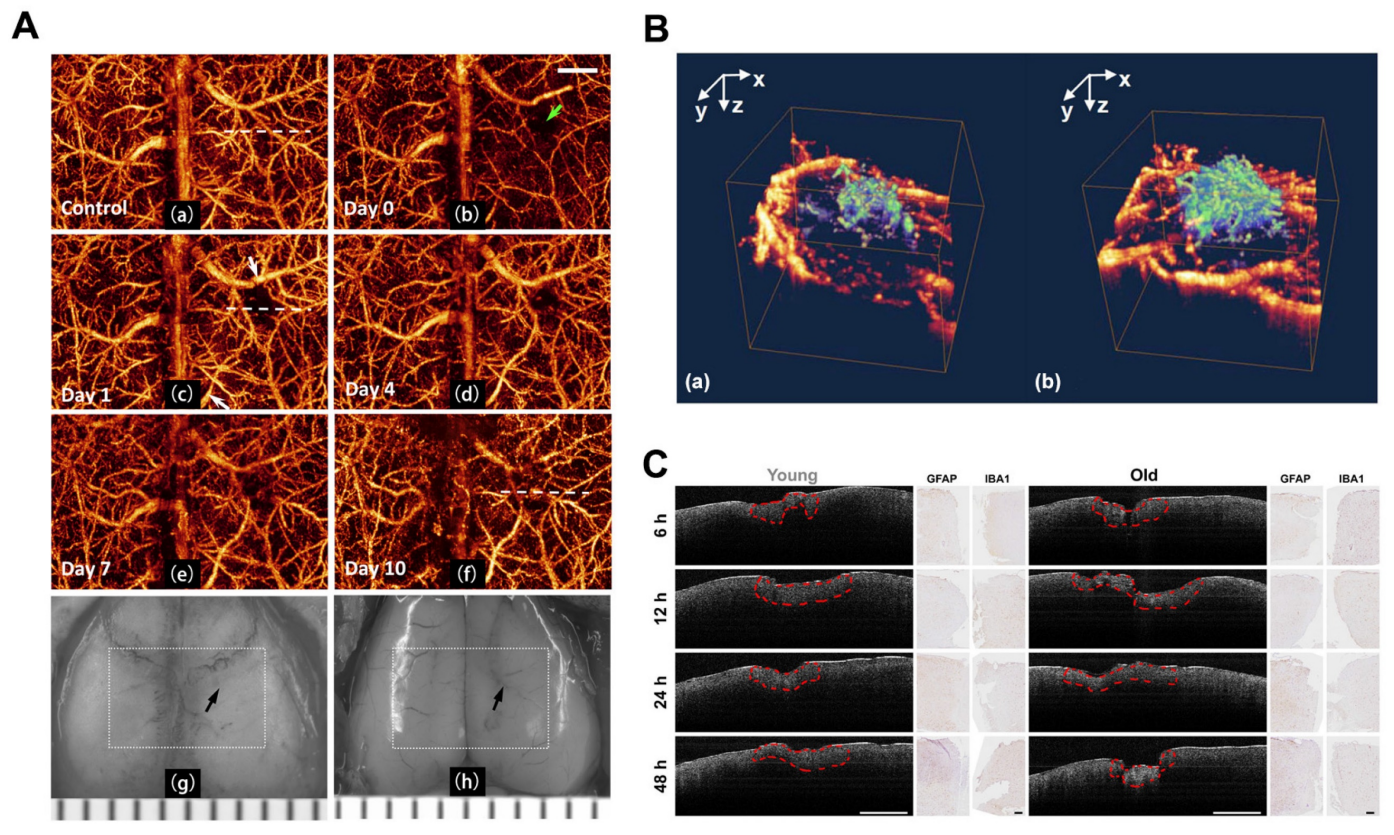
**(A)** Continuous 3D OMAG imaging of the cortex during TBI in mice. Compared to baseline, vascular remodeling and neovascularization occurred gradually in the trauma area during TBI recovery. Reproduced with permission from 94, copyright 2009 SPIE. **(B)** 3D OMAG images of the mouse brain at week 4 after trauma show vascular reconstruction (green) at the wound site and surrounded by undamaged functional blood vessels (yellow). Reproduced with permission from 95, copyright 2011 Elsevier. **(C)** OCT scan and histological findings in the acute phase of TBI. Examples of OCT areas analysis are highlighted with red dashed line for both young and old animals, while the histological optical picture is presented for astrocytes (GFAP) and microglia/macrophages (IBA1) at the same time points. Reproduced with permission from 99, copyright 2021 Wiley.

With the development of the OMAG system 26, Wang *et al.* initially designed a high-speed and high-sensitivity OMAG imaging system to obtain high-quality *in vivo* cerebrovascular blood perfusion imaging on intact skin and skull with resolution reaching the capillary level. This system effectively eliminated interference from skin surface blood perfusion 93. Three years later, Wang *et al.* demonstrated the ability of OMAG to perform repeat imaging of the three-dimensional cerebrovascular system in the pre-trauma and post-trauma phases. They effectively visualized changes in CBF regulation and vascular plasticity after trauma (**Figure 7A**) 94. The researchers also utilized optical microangiography (OMAG) to investigate endogenous revascularization in live mice post-brain injury. Additionally, they assessed the impact of pharmacological agents that either hinder or facilitate endogenous revascularization in the recovery phase of small rodent models (**Figure 7B**) 95.

As older animals exhibit severe neurodegeneration compared to younger animals 96-98, prolonged edema during the acute phase of injury increases the disruption of the blood-brain barrier. Osiac *et al.* distinguished the subtle morphological changes that arise in the brains of young and old mice during the acute phase of TBI by employing OCT techniques 99. They compared OCT brain images of young animals with TBI against those of older animals exhibiting similar lesions. Furthermore, they investigated the capability of OCT imaging in identifying the diffuse morphological changes associated with TBI during the chronic phase. The OCT imaging sessions were conducted 7, 14, 21, and 30 days post-TBI (**Figure 7C**).

Although TBI and stroke are distinct conditions, TBI may potentially precipitate stroke occurrences. Hemorrhagic strokes commonly arise from TBI, as the vascular damage induced by TBI disrupts blood vessels, causing rupture and subsequent bleeding. TBI may also trigger thrombosis, potentially leading to an ischemic stroke 100-102. In addition, stroke increases the risk of traumatic brain injury, resulting in notably elevated mortality rates in stroke patients with coexisting TBI, particularly those hospitalized post-TBI. The severity of stroke is correlated with mortality following TBI. Since both stroke and TBI offer insights into neuronal injury and neuroprotective mechanisms, including evaluating the impacts of diverse medications and treatments on neuronal preservation and functional restoration, both models have advanced alongside progress in Optical Coherence Tomography (OCT) technology and stand to mutually benefit from each other.

## OCT Imaging of Brain Cancer

Brain cancers are malignant tumors that develop within the human brain or its adjacent tissues, posing a significant threat due to their presence in the vital organs of the body. The exact etiology of these cancers remains incompletely understood; however, certain factors, such as age, genetic predisposition, exposure to specific radiation and chemicals, head trauma, and compromised immune function, are believed to heighten the risk of brain cancer development 103, 104. These cancers are commonly categorized into primary and secondary types. Primary brain cancers originate in the brain tissue, typically giving rise to one or more tumors. While primary brain tumors are often malignant, they may also manifest as benign. On the other hand, secondary brain cancers arise when cancerous cells from other regions of the body metastasize to the brain via the bloodstream or lymphatic system, forming cell clusters within the brain. Secondary brain cancers are typically more prevalent than primary brain tumors.

In brain cancer, OCT technology serves as a valuable tool for the quantitative assessment of morphological and functional changes in brain tissue, aiding in diagnosing and evaluating brain tumors. OCT allows real-time visualization of brain tissue structures and lesions by generating high-resolution tomographic images. OCT technology plays a crucial role in the diagnosis of brain cancer by enabling physicians to observe and assess pathological changes in brain tissue, including vascular proliferation, alterations in cell density, and disruptions in tissue structure. These observations are instrumental in delineating the extent of cancerous regions and informing treatment strategies. Furthermore, the synergistic application of OCT technology with other imaging modalities enhances the precision of pathological localization and diagnostic accuracy in cases of brain cancer.

Malignant gliomas represent the predominant form of brain tumors, constituting 63% of all astrocytic tumors 105. A defining characteristic of these tumors is their unique ability to infiltrate the white matter surrounding the brain, resulting in an indistinct boundary between the tumor and the brain tissue. The prognosis for patients with malignant gliomas is heavily reliant on the morphological and molecular genetic traits of the tumor. Glioma surgery aims to maximize tumor resection while minimizing damage to critical functional regions of the brain 106-109. However, conventional tumor extraction procedures using white light microscopy achieve maximal resection in only 23%-50% of cases 110, 111. In the imaging and treatment of malignant gliomas, OCT-related systems offer a promising approach for precise identification and treatment. Utilizing OCT-based diagnosis with intraoperative histological sections enables real-time differentiation between tumor tissue, non-tumor tissue, and infiltrated areas.

**Figure 8:**
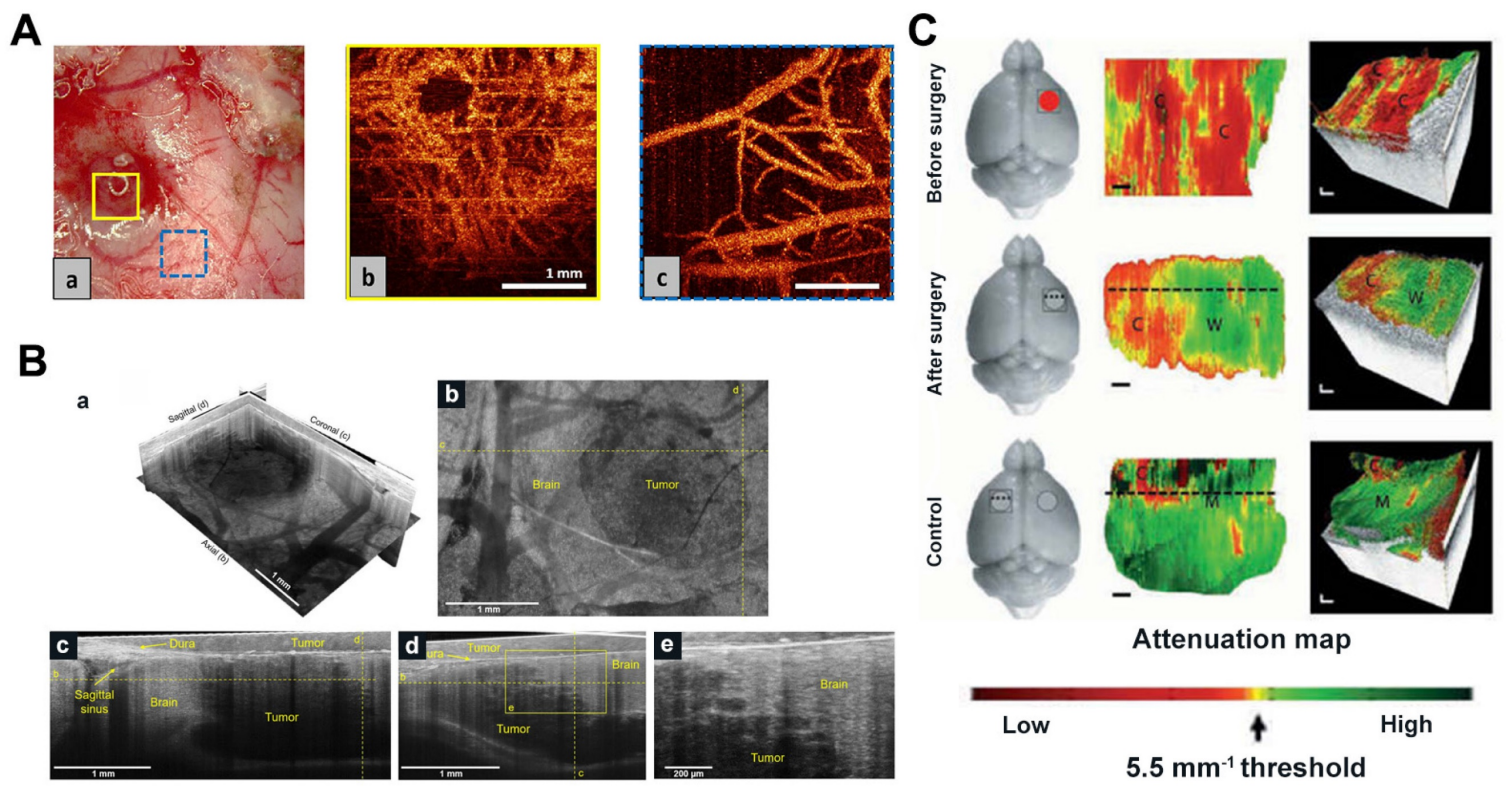
** (A)** OCT microangiography of (a) glioblastoma microvessels and (a) normal cortical vessels near tumor nodules in rats. Reproduced with permission from 114, copyright 2017 SPIE. **(B)** SM-OCT reveals high-resolution features of mice tumor margin *in vivo*. (a) SM-OCT ortho-slice of the tumor volume, showing the different sections in three dimensions. (b) SM-OCT axial view of mouse cortex with a GBM tumor. (c,d) SM-OCT coronal and sagittal views, respectively, showing the tumor margin. (e) A close-up view of the tumor margin in (d), showing the finger-like invasion of the into the surrounding brain tissue. Reproduced with permission from 122, copyright 2019 nature portfolio. **(C)** *In vivo* imaging of mice after resection of brain cancer. Representative results were shown for healthy areas of the mouse brain before (a), after (b), and on the opposite left side of the brain (c), respectively. The red circle represents the cancer, the gray circle represents the excision cavity, and the square represents the OCT FOV. Reproduced with permission from 116, copyright 2015 Science Translation Medicine.

In 2009, an article demonstrated the capability of OCT to differentiate normal brain tissue, diffusely infiltrating brain tissue, and solid tumors. Böhringer *et al.* used OCT to image human glioma biopsy specimens, revealing its efficacy in performing 2D and 3D optical tomography of the tumor-brain interface. Their study highlighted that the microstructure of the analyzed tissue and its light attenuation properties can differentiate between normal brain tissue, tumor-infiltrated regions, solid tumors, and necrotic areas 112, 113. Researchers have recently employed techniques like OCT and OCTA to observe brain tumor progression and discern variations between normal and malignant brain tissue (**Figure 8A**) 114, 115.

Kut *et al.* used attenuation coefficients for the first time to distinguish tumor tissue from white matter (**Figure 8C**) 116. The use of OCT attenuation maps demonstrated the practical potential of OCT to accurately distinguish cancerous and non-cancerous tissue in rat brain neurosurgery. Furthermore, Kut *et al.* introduced a groundbreaking artificial intelligence (AI)-assisted technique in another intraoperative OCT study, enabling the automatic detection of non-cancerous brain tissues infiltrating gliomas. This method is based on OCT and facilitates the real-time identification of glioma infiltration with high spatial resolution 117. Yashin and Achkasova *et al.* also used cross-polarized OCT (CP-OCT) to distinguish tumor and non-tumor tissue in human brain tissue. The visual assessment of structural CP-OCT images can detect areas of white matter with damaged myelinated fibers and distinguish them from normal white matter and tumor tissue 118-120. Attenuation coefficients are effective in distinguishing among different types of brain tissue, with damaged myelinated fibers exhibiting significantly lower attenuation coefficient values compared to healthy white matter.

Integrating various OCT modalities with conventional imaging techniques allows for a multimodal approach to imaging brain tumor microstructure, brain oxygen transport, and energy metabolism. Yaseen *et al.* developed a multimodal imaging system including two-photon and confocal microscopy, optical coherence tomography, laser scattering imaging and optical intrinsic signal imaging to investigate various aspects of cerebral blood flow and metabolism in small animal models 52. Zhu *et al.* devised an accurate point-to-point alignment-based bimodal optical diagnostic method for distinguishing brain tumors from normal tissue in neurosurgery 121. Quantitative autofluorescence spectroscopy and OCT were used to establish a cohort of brain tumor mouse models and to provide preoperative information using bioluminescence imaging. In 2019, Yecies *et al.* proposed a new neuroimaging technique, spot-modulated OCT (SM-OCT), which demonstrated exceptional resolution and a wide field of view in imaging living mouse brains and isolated human samples using only endogenous contrast. SM-OCT effectively identified brain tumor boundaries with a remarkable resolution of approximately 10μm (**Figure 8B**). The enhanced visibility resulting from speckle elimination reveals the white matter tracts and cortical layer structures of the living mouse brain, aligning well with the histological findings 122.

In recent years, intraoperative OCT has gained significant attention in brain tumor research. Finke *et al.* introduced a novel approach by integrating OCT with a robotically controlled surgical microscope, showcasing the automated acquisition of OCT images across the entire resection cavity. The fusion of microscope images with depth data from OCT enhances the identification of residual tumor cells 123. Various researchers have developed specialized OCT neurosurgical probes for visualizing brain tissue. One notable advancement is the handheld OCT imaging probe, designed with a bayonet profile akin to commonly used non-imaging Doppler ultrasound probes 124. The handheld bayonet-shaped design of OCT facilitates imaging of internal tissue microstructures previously inaccessible to surgeons, potentially establishing it as a potent imaging modality for surgical guidance. Chang *et al.* proposed an innovative diagnostic and therapeutic strategy employing a desktop SD-OCT combined with a high-power laser, creating a compact integrated platform for precise brain tumor resection 125. Fan introduced a novel system integrating SD-OCT with laser ablation therapy for the excision of soft biological tissues. OCT feedback guided the ablation process on *in vitro* porcine brain tissue samples 126. Katta lately presented an image-guided laser surgical system that successfully removed tumors *in vivo* within a mouse model of brain cancer xenotransplantation. This system offers marker-free imaging of vasculature and margins, facilitating precise coagulation and bloodless tumor removal 127.

In the future, the integration of morphological and functional data in the imaging of brain tumors is anticipated to significantly enhance the clinical utility of OCT in brain science.

## Extension of OCT imaging in clinics

OCT, as a high-resolution, high-speed, cost-effective, and non-invasive nature with no radiation exposure, has been widely used in preoperative diagnosis and intraoperative guidance, especially in the detection of eye diseases 128-133. The large number of recent studies based on OCT have also expanded the functionality of OCT even further. For example, Zhou *et al.* present 3D optical coherence refraction tomography (OCRT) to form a resolution-enhanced, speckle-reduced, refraction-corrected 3D reconstruction 134. Zhao *et al.* develop a spatially multiplexed phase pattern which successfully extended the DOF of our optical coherence tomography (OCT) system 135. Winetraub *et al.* developed a micro-registered OCT that can take a two-dimensional (2D) H&E slide and find the exact corresponding section in a 3D OCT image taken from the original fresh tissue 136. OCT research has expanded to encompass neurosurgery and brain diagnosis and treatment in recent years 137-139, and has been well integrated with deep learning 140-143. In clinical practice, OCT showcases the ability to discern biological tissue structures at the micron scale, with functional OCT demonstrating significant clinical research and application potential, including nerve fiber bundles and neurovascular imaging. OCT can identify tumor edges, offering valuable intraoperative guidance for tumor resection. Moreover, the use of OCT in therapeutic diagnostics is emerging as a promising approach in preclinical neurosurgical procedures, exemplified by its integration with laser ablation techniques 144-146.

Numerous studies have validated the efficacy of OCT in enhancing the precision of neurosurgical tumor resections by enabling high-resolution tumor identification. Bizheva *et al.* exhibited the capabilities of ultra-high-resolution OCT (UHR OCT) on *ex vivo* human tissues in delineating crucial morphological features like microcalcifications (> 20 μm), enlarged nuclei of tumor cells (similar to 8 to 15 μm), small cysts, and blood vessels that are indicative of neuropathologies and typically absent in healthy brain tissue 147. Based on microstructure and B-scan signal characteristics, Böhringer *et al.* demonstrated that SD-OCT can differentiate between solid brain tumors, diffusely invaded brain tissue, and adjacent normal brains. With its rapid image acquisition rates, SD-OCT technology shows promise as an innovative intraoperative imaging tool for detecting residual tumors and guiding neurosurgical tumor resections 113.

**Figure 9:**
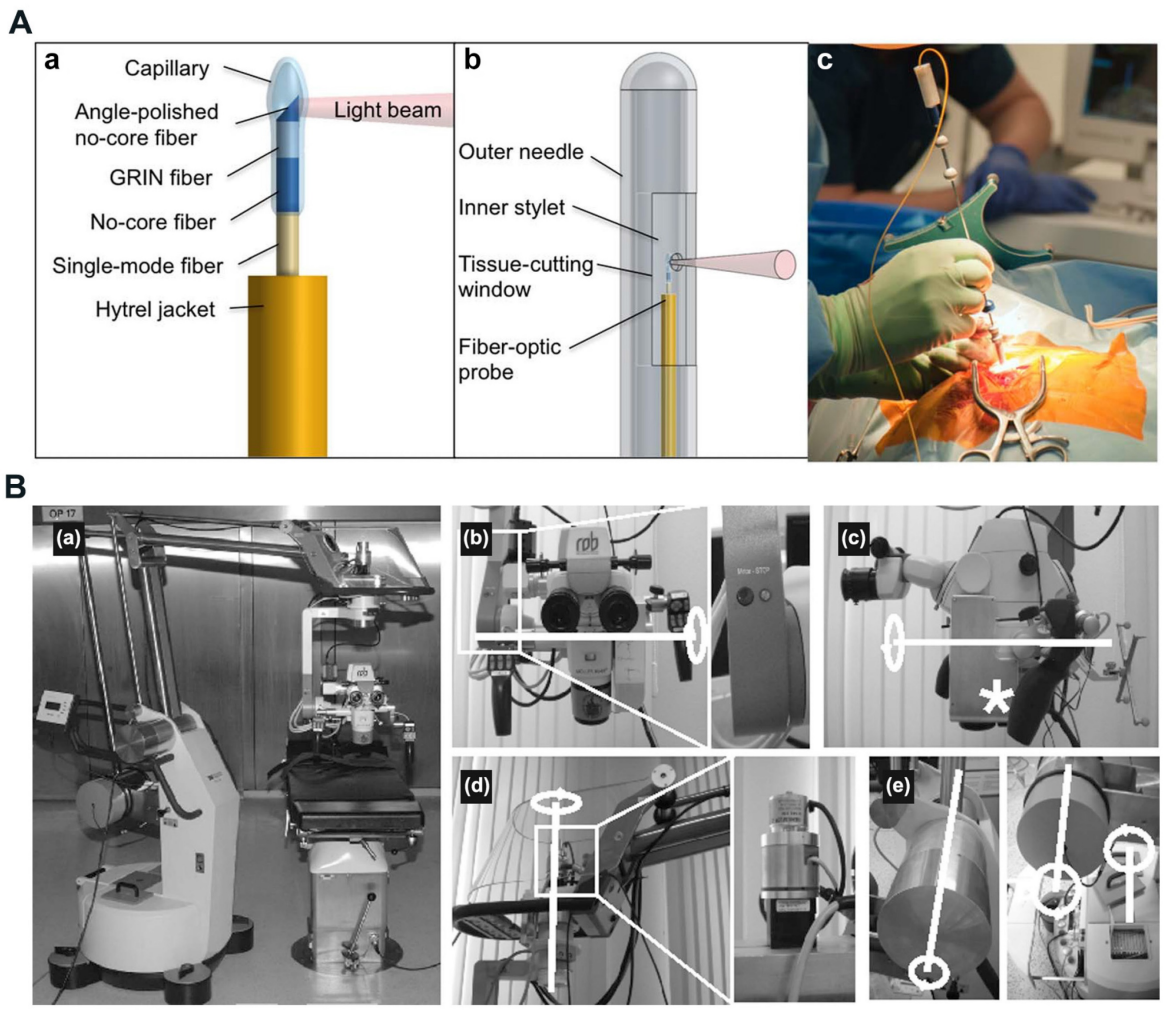
** (A)** Imaging needle design. (a) Schematic of the distal end of the fiber-optic probe. (b) Schematic of the distal end of the imaging needle, showing the outer needle, inner stylet, and fiber-optic probe. (c) Photo showing the imaging needle inserted into a human brain during surgery. Reproduced with permission from 151, copyright 2018 Science Advances. **(B)** The completely robotized Möller-Wedel Hi-R 1000 microscope, which retains full manual control of the conventional auto balanced microscope (a). (b)through (e), the internal and external motors, gearboxes, and encoders (details in the magnified boxes) and the motorized axes (highlighted in white). Optical coherence tomography-scanning unit integrated into the light path of the microscope (asterisk in c). Reproduced with permission from 155, copyright 2013 Neurosurgery.

OCT proves effective in identifying isolated tumor areas and serves as a valuable tool in intraoperative diagnosis during neurosurgery. The high speed and resolution of OCT, combined with the integration of multimodal systems, enhance the accuracy and efficiency of detection and diagnosis. Various researchers have innovatively designed and fabricated specialized neurosurgical OCT probes, such as endoscopic, needle-type, hand-held probes, and robotic arms, to provide detailed insights into brain tissue 146. Boppart *et al.* constructed a portable, handheld OCT surgical imaging probe to assess the utility of OCT as a high-resolution, real-time intraoperative imaging modality for detecting intracortical melanoma. The two-dimensional image results based on the cadaveric human cortex with metastatic melanoma revealed increased optical backscattering within the tumor region, facilitating quantitative determination of the tumor boundary. These images were consistent with the histological findings 148. Böhringer *et al.* utilized a Sirius 713 OCT device to a modified rigid endoscope and showed that OCT integrated endoscope can image the endoventricular anatomy and other endoscopically accessible structures in a human brain specimen 149. Sun *et al.* developed a prototype neurosurgical hand-held OCT imaging probe to provide micron-resolution cross-sectional images of subsurface tissue during open surgery 150. Ramakonar *et al.* pioneered using an optical coherence tomography needle probe in the human brain *in vivo*. They successfully developed an "imaging needle" equipped with a miniaturized optical coherence tomography probe capable of real-time visualization of nearby blood vessels (**Figure 9A**) 151. Yan *et al.* developed a flexible sensorized robotic OCT neuroendoscope, which combines a 2-degree-of-freedom (DOF) cable-driven continuum manipulator (CM) with an ultrahigh-resolution 800-nm OCT probe and a multicore fiber Bragg grating (MCFBG) fiber sensor, and demonstrated its clinical potential for minimally-invasive imaging-guided diagnosis and treatment in deep brain *in vivo* 152.

Integrating OCT imaging with an operating microscope enhances visualization of the surgical area. Microscope-assisted OCT-based neurosurgical guidance offers improved resolution and a wider field of view for precise surgical procedures 146. Lankenau *et al.* combined OCT with an operation microscope to enable real-time, non-contact OCT imaging across various medical procedures. This innovative approach allows for visualization of cochlear morphology without the need to open enveloping membranes 153. Guo *et al.* reported a robust wearable OCT probe, which is the first wearable OCT angiography probe capable of long-term monitoring of mouse brain blood flow 154. Kantelhardt *et al.* assessed a roboticized operating microscope prototype with an integrated optical coherence tomography module (**Figure 9B**). Their findings suggest that future advances in operating microscopes may enable the acquisition of intraoperative spatial data, volume changes, and structural details of brain or brain tumor tissue 155. Seong *et al.* developed a virtual intraoperative OCT angiography integrated surgical microscope (VI-OCTA-SM) to simultaneously visualize morphological tissue structure and microvasculature data of the surgical region including tumor margin and blood vessel map, which demonstrated its potential in neurological surgeries 156.

In summary, intraoperative brain imaging based on OCT can provide sufficient structural and functional information for precise clinical guidance. In the future, OCT has even greater advantages and potential for ultra-high-resolution brain imaging, neurosurgical surgical guidance, and minimally invasive therapeutics in combination with laser ablation.

## Challenges

As an emerging optical technology, OCT can be applied in clinical practice because of its multiple advantages, such as non-invasive, radiation-free, and real-time observation of fine structures around lesions with high resolution. Moreover, OCT-based blood flow imaging is widely used for monitoring vascular networks. However, numerous *in vivo* studies have demonstrated the imaging capability of OCT, the clinical application of OCT in the brain is still limited compared with other established imaging modalities, such as MRI and CT, etc. The main limitation of OCT is the limited depth of imaging in biological tissues. In general, the penetration depth of OCT is limited to 2-3 mm, which is suitable for light-transmitting ocular tissues or superficial tissues, but not sufficient to penetrate the skull. Additionally, OCT presents a restricted field of view (FOV), and in experiments, angiography struggles to accurately measure dimensions less than 20 μm 17. Furthermore, motion-induced noise has the potential to significantly degrade the quality of OCTA. Consequently, the primary focus of OCT advancement is to enhance imaging depth and FOV while reducing motion artefacts. The system in OCT-guided neurosurgical theranostics also needs higher technical advancements for faster automatic diagnosis and therapy 157,158 as well as fusion of multimodal information 159,160.

One potential solution to the limitations of OCT is the integration of advanced adaptive optics 161,162 These optics automatically adjust the aberrations of the optical system based on the optical properties of the sample, thereby improving resolution and depth penetration by compensating for optical aberrations. It typically extends the imaging depth beyond the typical 2-3 mm range. Recent studies have also shown that needle-shaped beams can effectively extend the depth of focus of an OCT system, improving lateral resolution, signal-to-noise ratio, contrast and image quality over a long depth range 163. In addition, wavefront shaping technology corrects aberrations in the optical system, including scattering and aberrations, to significantly improve imaging quality 164,165. It also reduces the scattering and absorption of light in tissues, thereby increasing imaging depth. For problems with limited fields of view, scanning protocols that include a wider range of scanning angles can be used, thus covering a wider area during imaging. To minimize motion-induced noise, the use of a real-time tracking system that can be adjusted to the patient's movements can maintain image quality during the scanning process. In addition, image clarity and accuracy can be further enhanced with more powerful image processing software, including noise reduction algorithms designed specifically for OCT angiography 166,167.

With the continuous advancement of OCT and OCTA technologies, these non-invasive imaging methods will play an increasingly important role in the early diagnosis, disease course monitoring and personalized treatment of brain diseases. Recent studies have also shown that the application of OCTA to explore vascular abnormalities in other neurological disorders (*e.g.*, epilepsy, autism) 168, providing new perspectives for disease mechanism studies. In the future, through interdisciplinary cooperation and technological innovation, it is expected that the depth and breadth of the application of these technologies will be further enhanced, bringing new breakthroughs in neuroscience research and clinical practice.

## Conclusions

Based on these findings, OCT is a promising and rapidly evolving approach that fills the gap between classical imaging studies (MRI and ultrasound) and new subcellular resolution methods such as multiphoton imaging. Unlike MRI or CT, OCT uses near-infrared light sources without the risk of tissue damage, does not require injections or expose patients to ionizing radiation, and allows for real-time, non-invasive imaging. In addition, OCT has a higher resolution (~10 microns) than other optical imaging methods such as photoacoustic imaging or speckle imaging. In addition, it can be integrated into operating microscopes or endoscopes to improve visualization of more imaged areas. As technology advances, OCT can be integrated with other systems and advanced algorithms to increase the available depth of imaging (~3mm) and provide more functional parameters, which improves the intelligence of diagnostic and therapeutic systems.

In summary, OCT has a broad range of clinical applications in the brain in the future, and its potential to produce qualitative and quantitative brain representations is evident. We describe the ability of OCT to image changes in the structure and blood flow of brain tissue and describe its function in stroke models, traumatic brain injury models, and brain cancer models. It's expected that the clinical diagnostic capabilities of OCT will continue to improve in the near future, allowing for rapid and effective brain detection during surgery. Finally, we foresee OCT and OCT angiography is anticipated to become a valuable tool for high-precision, automated, and intelligent clinical brain diagnosis and treatment.

## Figures and Tables

**Table 1:** Summary of the application of OCT in brain disorders

Brain disorders	Authors	Systems	Models	Measurement
Ischemic stroke	Y. Jia, R. K. Wang. 77	OMAG imaging system	Ischemic stroke model	3D distribution of dynamic blood perfusion
Ischemic stroke	V. J. Srinivasan *et al.* 78	Spectral/Fourier domain OCT	fMCAO and dMCAO	Brain injury progression
Ischemic stroke	Yang S *et al.* 79	Gaussian beam OCTA	focal photothrombosis stroke model	Dynamic changes of blood vessels and tissues
Ischemic stroke	W. J. Choi, Y. Li, R. K. Wang. 81	Single, integrated imaging platform	dMCAO	Changes in blood perfusion, blood flow, erythrocyte velocity, and light attenuation
Brain Injury	R. K. Wang, S. Hurst. 93	OMAG imaging system	TBI	High-quality *in vivo* cerebrovascular blood perfusion imaging on intact skin and skull
Brain Injury	E. Osiac *et al.* 99	SSOCT	TBI	Cortical changes in the acute phase of a penetrating TBI
Brain cancer	C. Kut *et al.* 116	Label-free, quantitative SSOCT	*Ex vivo* human brain tissues	Classification of different grades of human brain cancer from noncancer
Brain cancer	M. Zhu *et al.* 121	Dual-modality system	Mouse brains with human glioma cells	Both biochemical information and structural information
